# Decoding the Neurodevelopment and Seizure Puzzle: A Pediatric Case of DYRK1A Gene Mutation and Autosomal Dominant Mental Retardation Type 7

**DOI:** 10.7759/cureus.57460

**Published:** 2024-04-02

**Authors:** Abdulrahman A Aldoseri, Rashed N Buhaza, Raafat Hammad Seroor Jadah

**Affiliations:** 1 Pediatrics, Bahrain Defense Force Hospital, Riffa, BHR; 2 Paediatrics and Child Health, Bahrain Defense Force Hospital, Riffa, BHR

**Keywords:** epilepsy, dyrk1a, seizure, chromosome, gene mutations, neurodevelopment

## Abstract

Autosomal Dominant Mental Retardation Type 7 is a disorder caused by pathogenic variants in the *DYRK1A *gene. Clinical features associated with this gene mutation include focal dysmorphism, developmental delay, and epilepsy. In this report, we present a case of an 8-year-old boy with a *DYRK1A* gene mutation, whose clinical manifestations underscore the rarity and clinical challenges of this genetic condition. The patient is a known case of global developmental delay with intractable epilepsy on multiple anti-epileptic medications. Upon examination, the patient showed delayed developmental milestones, hypotonia with brisk deep tendon reflexes, as well as dysmorphic features in the form of microcephaly, deep-set eyes, prominent ears, and a short nose. MRI was done, and findings were suggestive of a *DYRK1A *gene mutation. The diagnosis was later confirmed by Whole Exome Sequencing (WES). Our report aims to contribute to the growing knowledge about *DYRK1A* mutations, facilitating a better understanding of the associated clinical features and implications for patient care.

## Introduction

Within the landscape of rare genetic disorders, the *DYRK1A *gene mutation is a rare genetic disorder with only 68 cases has been reported in the literature leading to Autosomal Dominant Mental Retardation Type 7 with strikingly uncommon occurrence [1]. Manifesting in dysmorphic features, intellectual delay, and invariable form of epilepsy [2], this genetic anomaly has been identified in only a handful of cases worldwide. Insights gained from these cases enable healthcare providers to comprehend the intricate interplay between genetic factors and neurodevelopment disorders. The possibilities of *DYRK1A *gene mutation should be strongly considered in pediatric patients who present with such phenotype associated with this rare genetic disorder.

Following the confirmation of diagnosis by genetic testing, genetic counseling plays a crucial role in guiding families through the complexities of genetic testing and inheritance patterns [2]. By raising awareness of this rare genetic disorder, healthcare professionals can improve diagnostic accuracy and facilitate timely interventions for affected individuals.

## Case presentation

This is an eight-year-old boy, with a known case of global developmental delay (GDD) with intractable epilepsy on multiple anti-epileptic medications including levetiracetam, topiramate, and oxcarbazepine. This patient initially presented at the age of eleven months with frequent typical febrile convulsions, which later became an atypical form of febrile convulsion due to the long duration of the seizure, which lasted for more than 30 minutes. The patient also showed a progressive delay in his developmental milestones as he started to sit without support at the age of ten months and started to walk at the age of three years with poor verbal communication in the form of speech delay, saying only two to three words at the age of three and half years. He is a product of term delivery to non-consanguineous parents with no postnatal complications. No family history of epilepsy or seizure disorder.

Neurological examination showed an active child with poor cognitive and verbal communication and dysmorphic features in the form of microcephaly, deep-set eyes, prominent ears, and a short nose. Skin examination was negative for neurocutaneous stigmata. He is hypotonic with brisk deep tendon reflexes, however, no clonus, with normal power. Cranial nerve examinations were normal with no cerebellar signs. The gait examination was normal and the rest of the systemic examinations were unremarkable.

**Figure 1:**
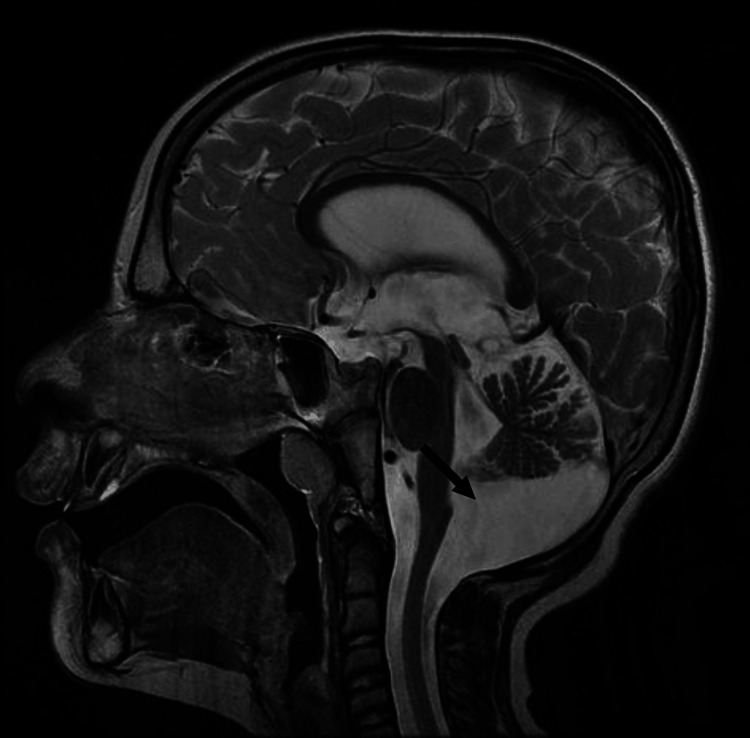
Enlargement of posterior fossa and 4th ventricle.

Magnetic resonance imaging (MRI) showed posterior fossa and 4th ventricle enlargement in sagittal T2 sequence images (Figure 1). 

**Figure 2:**
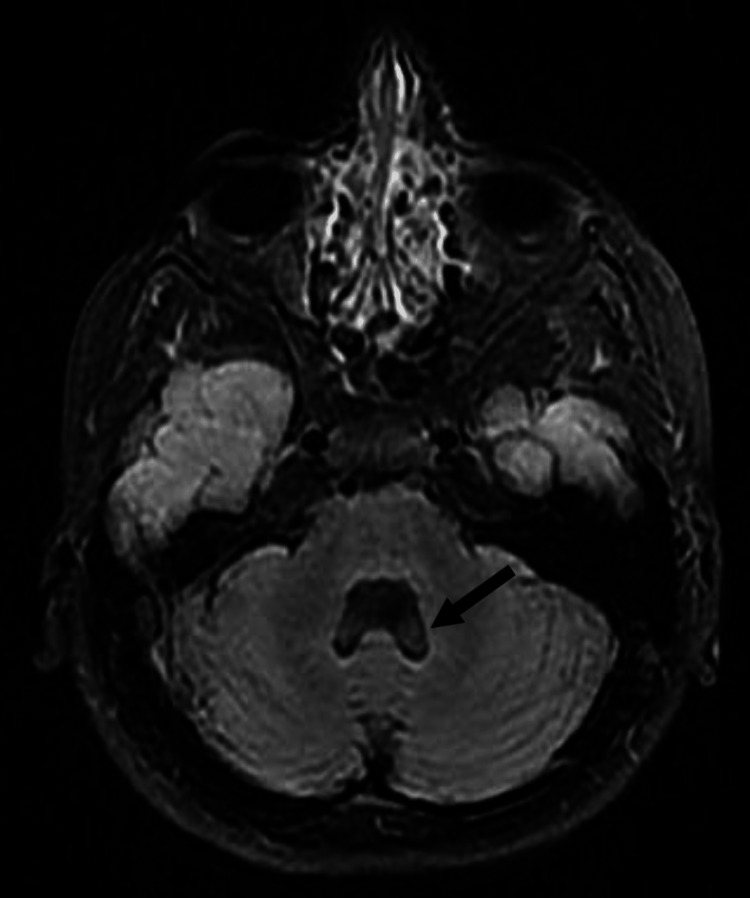
Enlargement of the 4th ventricle with vermis hypoplasia.

**Figure 3:**
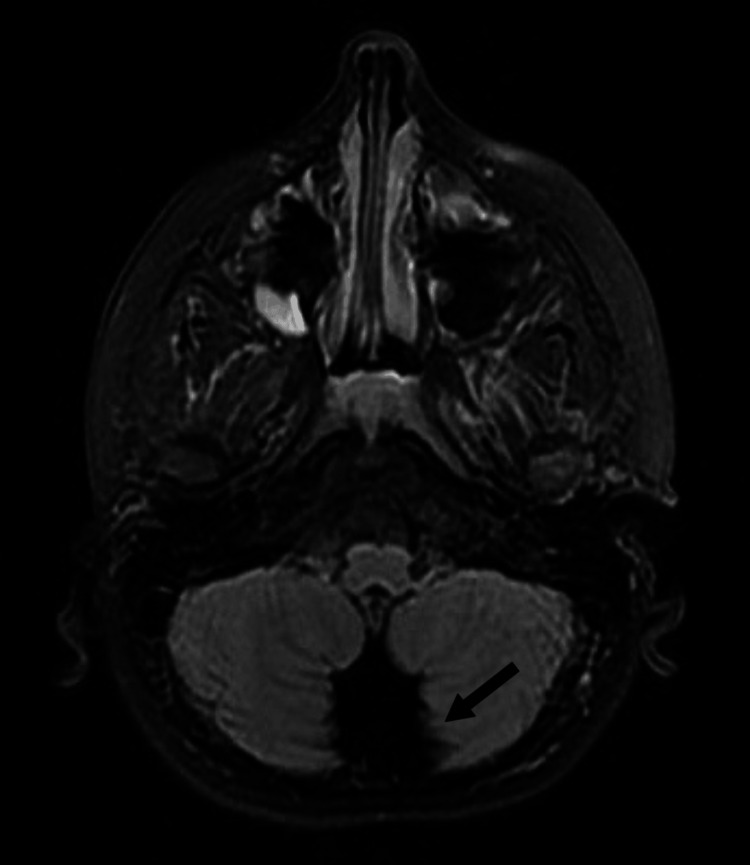
Posterior fossa enlargement with vermis hypoplasia.

Axial T2 fluid-attenuated inversion recovery (FLAIR) images also showed severe vermis hypoplasia (Figures 2, 3), as well as enlargement of the 4th ventricle (Figure 2), and significant posterior fossa enlargement (Figure 3). 

The patient underwent whole exome sequencing (WES) test, and a heterozygous likely pathogenic variant was identified in the *DYRK1A *gene. 

The identified variant interpretation was as follows: The *DYRK1A *variant c.588dup p. (Leu197Serfs*12) creates a shift in the reading frame starting at codon 197. The new reading frame ends in a stop codon 1 positions downstream. It is classified as likely pathogenic (Table 1). 

**Table 1:** whole exome sequencing (WES) test result interpretation Variant annotation based on OTFA (using VEP v94). *AlignGVD: C0: least likely to interfere with function, C65: most likely to interfere with function; splicing predictions: Ada and RF scores. **Genome Aggregation Database (gnomAD), Exam Sequencing Project (ESP), 1000Genome Project (1000G) and CentoMD (latest database available). ***based on ACMG recommendations.

GENE	VARIANT COORDINATES	AMINO ACID CHANGE	SNP IDENTIFIER	ZYGOSITY	NI SILICO PARAMETERS*	ALLELE FREQUENCIES**	TYPE AND CLASSIFICATION***
DYRK1A	NM_001396.3: c.588dup	p.(Leu197Serfs*12)	N/A	heterozygous	PolyPhen: N/A, Align-GVDG: N/A, SIFT: N/A, MutationTaster: N/A, Conservation_nt: N/A, Conservation_aa: N/A.	gnomAD: -, ESP: - 1000 G: -, CentoMD: -.	Frameshift Likely pathogenic (class 2)

The result is consistent with a genetic diagnosis of Autosomal Dominant Mental Retardation Type 7. Genetic counseling was done, and parental carrier testing was recommended.

## Discussion

*DYRK1A*, located on chromosome 21q22.13, encodes a dual-specificity tyrosine-phosphorylation-regulated kinase, playing a pivotal role in neurodevelopment. This gene's mutations result in variable clinical presentations, contributing to the limited recognition of this rare condition [3]. 

Moreover, patients harboring *DYRK1A *mutations consistently exhibit developmental delays, speech impairments, and refractory epilepsy, as observed in our case. These manifestations highlight the neurological impact of *DYRK1A *on cognitive and motor functions, posing diagnostic and therapeutic challenges [4].

Our patient presented with dysmorphic features in the form of a beaked nose and micrognathia, with intractable epilepsy on multiple epileptic medications. Distinctive facial characteristics, to illustrate, a prominent forehead, beaked nose, micrognathia, and hypertelorism have been identified in several cases with *DYRK1A *mutations [5]. All in all, these dysmorphic features offer clinical clues, reinforcing the link between genetic abnormalities and phenotypic expressions. 

The conducted neuroimaging, such as MRI, often revealed cerebellar anomalies in *DYRK1A *cases. Hypoplasia of the inferior cerebellar vermis and the presence enlarged of cystic areas within the posterior fossa are recurrent findings [6]. These neuroimaging patterns enhance our understanding of the structural impact of *DYRK1A *mutations.

Our patient’s brain MRI showed features suggestive of vermian hypoplasia. Whole Exome Sequencing emerged as a pivotal tool in diagnosing rare genetic disorders, including *DYRK1A* mutations. This technique offers a comprehensive view of genetic variants, aiding clinicians in unraveling complex cases [7]. Hence, the patient presented in this case was diagnosed based on Whole Exome Sequencing.

## Conclusions

This case underscores the rarity and clinical complexity of *DYRK1A *gene mutations, contributing to the pool of global knowledge on Autosomal Dominant Mental Retardation Type 7. It presented with a typical clinical picture and was diagnosed by Whole Exome Sequencing, a diagnostic test. Through our report, we aim to fortify the medical community's awareness and appreciation of the intricate interplay between genetics and neurodevelopmental disorders.
